# Supplementary material for: Disentangling strictly self-serving mutations from win-win mutations in a mutualistic microbial community
Source: eLife. 2019 Jun 4;8:e44812. doi: 10.7554/eLife.44812 (PMC6548503; doi:10.7554/eLife.44812)
Supplement: Supplementary file 1. [file elife-44812-supp1.pdf]

Foresight, hindsight, insight, and the blur in-between

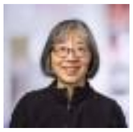

Wenying Shou

April 2019, Seattle, WA

Scientists prize foresight. Scientists value insight. Scientists dread hindsight—"In hindsight, we should have …" is a death sentence. I want to tell a story of resurrection: how insight could sometimes rise from the ashes of hindsight.

A well-written scientific research article is an exemplar of perfection. It starts with a compelling question. It is often driven by foresight—a logical or a radical hypothesis to address the question. It proceeds with hypothesis testing. It ends with insight—conclusions supporting (or refuting) the hypothesis. But hindsight, nearly worthless, is buried together with months or even years' toiling that led to it, leaving behind only regrets.

But is the distinction between hindsight and insight always that clear?

Can we catch win-win mutations evolving?

The story started with our fascination with cooperation. Imagine that you and I cooperate—that is, I help you and you help me back. Could a mutation that promotes self-interest serendipitously also shower more generosity toward the partner? We call such mutations "win-win" mutations. Essentially, by coupling self-interest with partner-interest, a win-win mutation promotes cooperation.

The existence of win-win mutations may require serendipity. After all, many mutants are "win-lose": benefiting self via exploiting partner. Interestingly, scientists have identified genes that couple self-interest with partner-interest during social amoeba cooperation (Foster et al., 2004) and during opportunistic pathogen cooperation (Dandekar et al., 2012). That is, a mutant that fails to help partner falls sick automatically and cannot survive long. Hence, win-win mutations are possible. However, these systems have had millions of years' history of cooperation, and no one has ever observed the emergence of win-win mutations in real time. So we asked: how easily can win-win mutations evolve?

To observe possible evolution of win-win mutations, we utilized an engineered community of two cooperating yeast strains. Each strain overproduces and releases a metabolite essential for the partner. We call this community CoSMO for "*Co*operation that is *S*ynthetic and *M*utually *O*bligatory" (Shou et al., 2007).

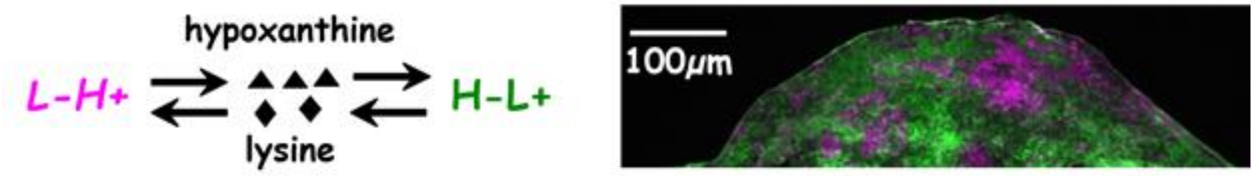

In CoSMO, two yeast strains exchange essential metabolites lysine (an essential amino acid) and hypoxanthine (which can be modified to make building blocks for DNA) (Hart et al., 2019). The two strains also express different fluorescent proteins, and can be visualized under a fluorescence microscope (Momeni et al., 2013).

The cooperative yeast community is great to work with. Besides the attractive bready and beery fragrance, yeast is a biologist's dream coming true. Lots of yeast cells can be grown in a short span of time, providing ample opportunities for mutations to occur and for evolution to show its hand. We can compare ancestral cells (the starting cells) with evolved cells (cells that had grown for a while and thus accumulated mutations). For example, we can compare their self-serving trait (e.g. how fast they grow) and partner-serving trait (e.g. how much metabolite they release for partner). We can identify mutations by sequencing and comparing DNA sequences of evolved cells with that of the ancestor. Importantly, CoSMO has *no* prior history of cooperation, so we could examine how easily win-win mutations could arise even if the two partners had never cooperated before.

Self-serving mutants in a "communistic" environment

Chi-Chun Chen, trained in physics and passionate about evolution biology, moved from Taiwan to join my lab as a research technician. Chi-Chun allowed multiple CoSMO communities to evolve by growing them in test tubes. Whenever a community grew to a light milky turbidity, he would dilute a small fraction of it into fresh medium. Chi-Chun continued this growth-dilution process for over a month, which spanned ~100 generations. During this, mutations would strike cells randomly, and fast-growing mutants would take over the culture.

If cells had grown on a nutrient pad, then nearby neighbors would interact with each other since released metabolites would stay around. Consequently, those who helped their cooperative neighbors would get more from neighbors and flourish, while those who did not help would eventually suffer as their neighbors perished. In contrast, in our evolution experiment, CoSMO communities were well-mixed. In an eerie sense, a well-mixed environment mimics a communistic society: every one's harvest is pooled, and the pooled harvest is then distributed evenly among members. Contrary to its utopian ideology, communism is known to lead to poverty: there is no incentive to help others, only incentive to help self. In the language of CoSMO, we would expect that all mutations would be self-serving.

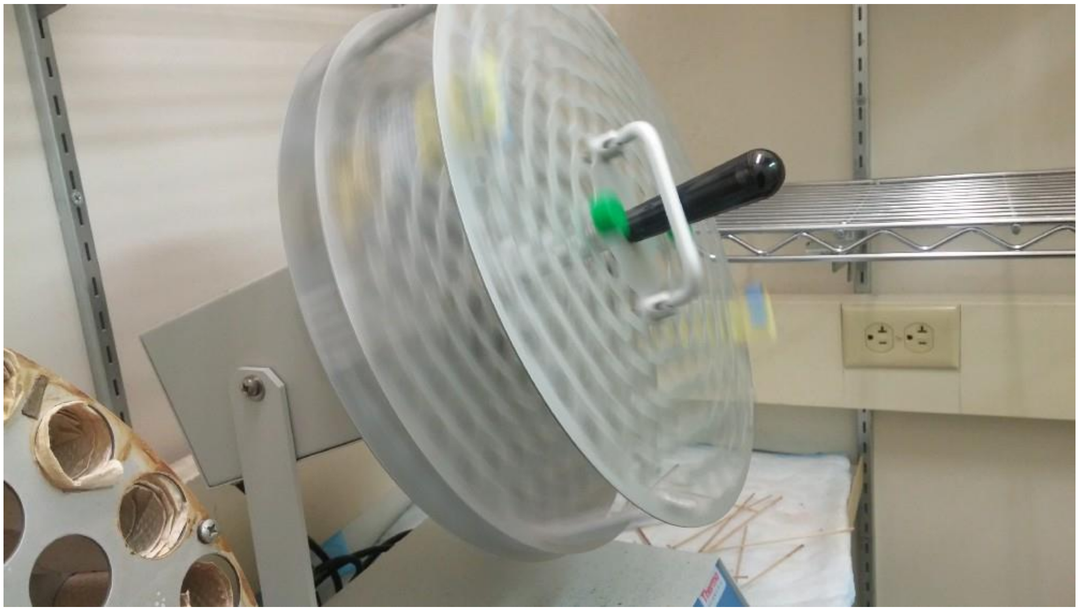
A roller drum keeps cultures well-mixed.

Indeed, every evolved cell we tested was better than the ancestor at competing for partner-supplied metabolite, and thus grew faster than the ancestor. Could some of these evolved cells happen to also be partner-serving? Intuitively, if a mutant cell has a higher metabolite release rate, it is more partner-serving, since the partner relies on the released metabolite.

Win-win mutation!

When Chi-Chun Chen left for graduate school, Jose Pineda took over the project. Jose was one of the very rare undergraduate students who could juggle many tasks efficiently. The 3-mile shuttle commute between our lab at the Hutch and Jose's classes at the University of Washington has dissuaded many undergraduates from continuing their research in our lab. Not Jose.

Strikingly, Jose found that some, but not all, evolved cells became more generous, each releasing more metabolite than the ancestor! All generous cells had an extra copy of Chromosome 14 (Chromosome 14 duplication). All other cells had the normal number of Chromosome 14. Thus, Chromosome 14 could harbor a "generosity" gene such that duplicating this gene made cells over-release metabolite.

We got really excited, especially since it was really easy to find win-win mutants harboring Chromosome 14 duplication. Everything also made so much sense: Sitting right within Chromosome 14 is the gene required for uptaking partner's metabolite. Thus, having an extra copy of Chromosome 14 is like growing an extra mouth, allowing the mutant to eat faster and thus grow faster than the ancestor. The generosity gene was more elusive, but still, the finding was cool!

In conferences, I talked about how we found Chromosome 14 duplication to be a win-win mutation, and people nodded. I then asked my Seattle yeast colleagues Maitreya Dunham and

Aimée Dudley, “As a reviewer, would you let us pass if we do not identify which gene on Chromosome 14 is the generosity gene?” They both answered, “No.”

Back to the bench. To finish this project, I encouraged Jose to take a year off and do full-time research as our research technician. I was very delighted when he agreed to it. It was an arduous journey. Jose started “chopping” off various segments of the duplicated Chromosome 14, using tricks provided by our yeast colleagues Dan Gottschling and Aimée Dudley. The logic is that if the chopped-off segment contained the generosity gene, then after chopping, the strain would no longer over-release metabolite. Chromosome 14 has about 400 genes, so Jose had to do multiple rounds of chopping.

Toward the end of that year, Jose was still far from wrapping up the project. Fortunately, Sam Hart joined our lab as a research technician. Sam was a very quick learner, taking over the project as Jose returned to finish his senior year at the University of Washington. Sam continued with chromosome chopping.

Not win-win…

The turning moment arrived.

I was at a Gordon Conference in the summer of 2015, and received an email from Sam, “Duplication of the *WHI*3 gene probably caused metabolite over-release.” He attached a power-point, with all the relevant information downloaded from the yeast genome database.

My heart sank. *WHI3* duplication is known to make cells bigger because *WHI3* duplication allowed cells to grow bigger before cell division (Nash et al., 2001). Since we had quantified generosity as the amount of metabolite released per hour per *cell*, a bigger cell could release more. However, a bigger cell might also consume more metabolites from the partner. In other words, the mutant may not be generous after all, if releasing more metabolite for partner requires consuming more metabolite from partner! In a short span of time, Sam showed that all my fears were correct.

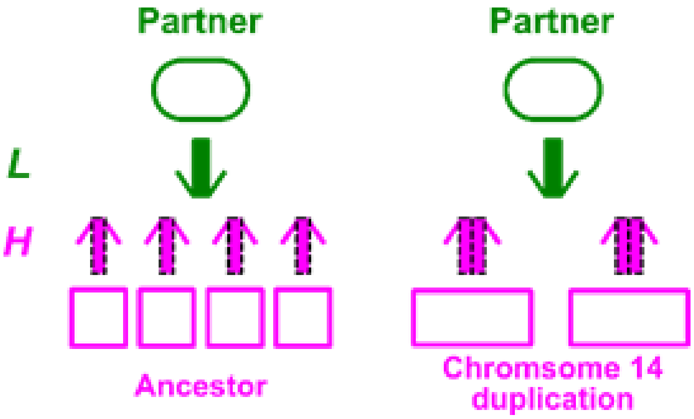

A bigger mutant cell releases more metabolite per cell, but also consumes more metabolite per cell. Thus, the mutant is not more generous than the ancestor.

The curse of hindsight

I could not forgive myself: "Wenying Shou! How could you have missed this? You are studying metabolite *exchange* between two populations. Why on earth would you only measure release, and not consumption??"

If I had the foresight, or rather, if I had the common sense, we would have measured the mutant's metabolite release and consumption, divided the two numbers, realized that the mutant was not win-win, and not wasted time chopping Chromosome 14… This hindsight was so trivial that I felt epically fooled.

I shoved this hindsight, together with two years' toiling by Jose and Sam, into a drawer. Of course, I was very grateful that yeast biologists had figured out what *WHI3* does; otherwise, we might still be struggling to figure out what *WHI3* does and/or publishing a wrong "win-win" paper. But this thought did not comfort me. I felt terrible—I had let my very excellent students down.

Resurrection

Our hindsight had taught us that generosity should be quantified as a ratio of release to consumption, which we termed "exchange ratio". Sam persisted, and measured the exchange ratios of scores of other evolved cells. To our delight, he found a hidden gem—a true win-win mutation!

As I started to write about the true win-win mutation, I found myself stuck. From the literature, it was not obvious that our initial criterion—increased release rate per cell—was inappropriate. Furthermore, details also mattered: why define exchange ratio as release *rate* : consumption *amount*, instead of release *amount* : consumption *amount*—like the foreign currency exchange ratio?

I then asked myself: is exchange ratio really hindsight? I recalled all the nods I received when giving my talks. I recalled the tendency in the literature where a lower-releasing mutant would be classified as "cheaters". Maybe, these were not cheaters—if they had consumed less.

I decided to pull the Chromosome 14 story back into the day light, and write it up as an independent paper. I told this story to my evolution biology colleague Harmit Malik at the Hutch, and he said, "Hmmm… this is a cautionary tale…" I then sent the abstract to Jim Bull, a well-known evolutionary biologist from UT Austin who has thought a lot about cooperation. Jim wrote back, "Wait—why can't you use the metabolite release rate to quantify partner-serving?" That was exactly what I had thought before *WHI3* smirked at me. My confidence was boosted.

On the last day of 2018, I submitted our manuscript to *eLife*, a journal with high bars. To my great relief, reviewers called our work "rather simple yet elegant". Reviewers asked for a more explicit comparison between our exchange ratio and the literature. After more literature and soul searching as well as checking with Kevin Foster and Ronald Noë (two additional experts on the

subject matter), I became even more convinced that our Chromosome 14 story offered us not hindsight, but insight. The paper (Hart, Pineda et al, 2019) is now accepted for publication.

In closure, if you should ever contemplate sentencing a hard-earned story to "hindsight", think again. Is the story really a hindsight and thus trivial? Or does it only seem trivial because you have made it so clear—an insight in disguise?

Acknowledgement

This story would not have been born without the three talented and devoted students from my lab.

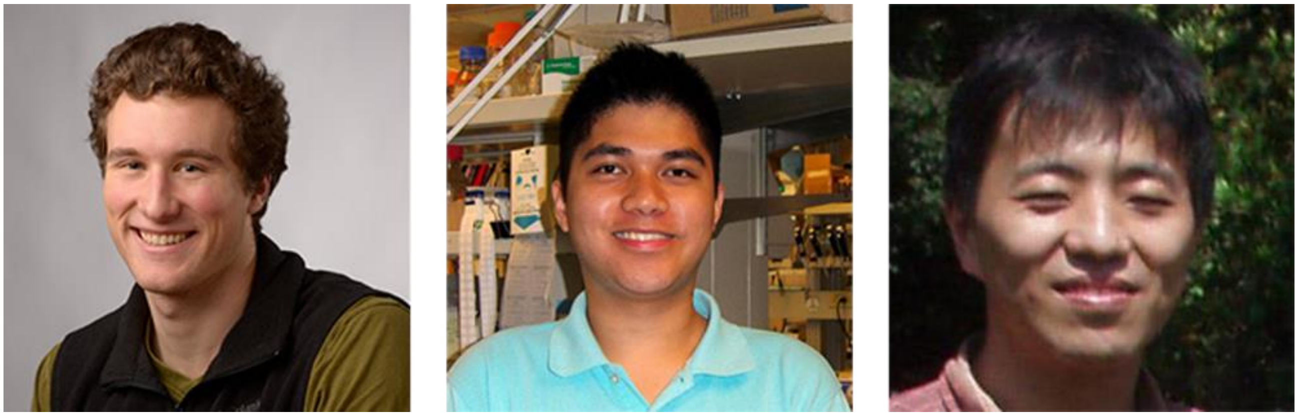

From left to right: Sam Hart, Jose Pineda, Chi-Chun Chen.

I am also grateful to Rachel Tompa, George Moore, Yan Fang, Sabrina Richards, and Michael Handelsman for critiques on my writing.

Notes

1. Underlines contain clickable links to articles.
2. For my other stories, see https://medium.com/@wenying.shou.
